# Supplementary material for: Identification of Freezing-Responsive microRNAs and Their Targets in Chinese Jujube by Small RNA and Degradome Sequencing
Source: Int J Mol Sci. 2026 Sep 4;27(17):7912. doi: 10.3390/ijms27177912 (PMC13566890; doi:10.3390/ijms27177912)
Supplement: Supplementary file 1 [file ijms-27-07912-s001.zip › Supplementary Material S2/novel_miR_214.pdf]

Provisional ID : novel_miR_214
Score total : 2392.6
Score for star read(s) : 3.9
Score for read counts : 2384.6
Score for mfe : 2.5
Score for randfold : 1.6
Score for cons. seed :
Total read count : 4689
Mature read count : 4541
Loop read count : 0
Star read count : 148

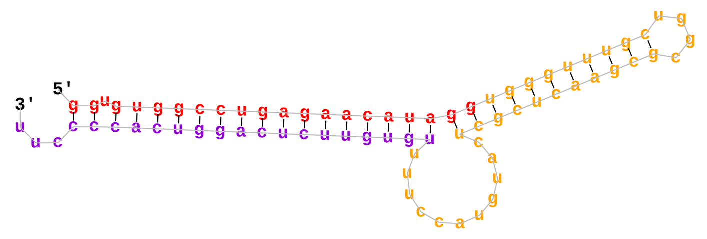

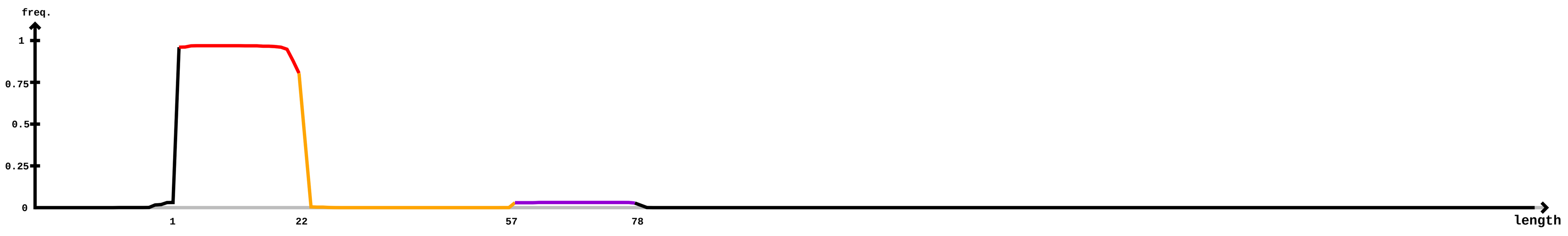


```
                          Mature                                          Star
5'- uuaugcagacgguucacccggagguguggccugagaacauagguggguuugcuggcgcgaacucgcucauguaccuuuuguguucucaggucaccccuuugggacacccucuguuuacaugcauauuuuauaugcacaaagcuuucucucucuauguaccuagacaaugcaaaguuaccaucuucuucuuuaagaacagcaauuguuuuuuuuuuaaacaaacugcaucugaaccguuggcuuaauccaa   -3'   obs
    uuaugcagacgguucacccggagguguggccugagaacauagguggguuugcuggcgcgaacucgcucauguaccuuuuguguucucaggucaccccuuugggacacccucuguuuacaugcauauuuuauaugcacaaagcuuucucucucuauguaccuagacaaugcaaaguuaccaucuucuucuuuaagaacagcaauuguuuuuuuuuuaaacaaacugcaucugaaccguuggcuuaauccaa         exp
    (((.((.((((((((((((((((.(((((((((((((((((((((((((((....))))))))))))..........))))))))))))))))).))))))))(((.....))).....(((((((...)))))))...((((((((....((((......))))....).))))))......(((((....)))))..(((.((((((.......))))))..)))...))))))))).)).))).....   reads   mm   sample
    ...........uucacccggaggugugg ccug.............................................   1   0   JCK-3
    ...........uucacccggagguguggccug..............................................   2   0   JCK-1
    ................ccggagguguggccugaga...........................................   2   0   LT-3
    .................cggagguguggccugaga...........................................   3   0   JCK-3
    .................cggagguguggccugaga...........................................   4   0   LCK-3
    .................cggagguguggccugaga...........................................   1   0   JCK-2
    .................cggagguguggccugaga...........................................   1   0   LT-2
    .................cggagguguggccugagaac.........................................   2   0   LT-1
    .................cggagguguggccugagaac.........................................   1   0   LT-2
    .................cggagguguggccugagaac.........................................   1   0   JCK-2
    .................cggagguguggccugagaac.........................................   3   0   LT-3
    .................cggagguguggccugagaac.........................................   2   0   JCK-3
    .................cggagguguggccugagaaca........................................   7   0   LT-2
    .................cggagguguggccugagaaca........................................   1   0   JCK-1
    .................cggagguguggccugagaaca........................................   4   0   LT-1
    .................cggagguguggccugagaaca........................................   4   0   LT-3
    .................cggagguguggccugagaaca........................................   2   0   JCK-3
    .................cggagguguggccugagaacau.......................................   1   0   LT-1
    .................cggagguguggccugagaacauU......................................   1   1   LT-3
    .................cggagguguggccugagaacaua......................................   2   0   JCK-3
    .................cggagguguggccugagaacaua......................................   2   0   LT-3
    .................cggagguguggccugagaacauag.....................................   1   0   LT-3
    .................cggagguguggccugagaacauagg....................................   2   0   JT-3
    .................cggagguguggccugagaacauagg....................................   3   0   LT-1
    .................cggagguguggccugagaacauagg....................................   5   0   JCK-3
    .................cggagguguggccugagaacauagg....................................   5   0   JCK-1
    .................cggagguguggccugagaacauagg....................................   9   0   JCK-2
    .................cggagguguggccugagaacauaggA...................................   3   1   JT-2
    .................cggagguguggccugagaacauaggA...................................   1   1   JT-3
    ..................ggagguguggccugagaac.........................................   1   0   LT-2
    ..................ggagguguggccugagaacau.......................................   2   0   JCK-1
    ..................ggagguguggccugagaacauag.....................................   1   0   JCK-2
    ..................ggagguguggccugagaacauag.....................................   2   0   JCK-3
```

Mature **Star**

uuaugcagacgguucacccgga**gguguggccugagaacauagg**ugggguuugcuggcgcgaacucgcucauguaccuuu**uguguucucaggucaccccuu**ugggacacccucuguuuacaugcauauuuuauaugcacaaagcuuucucucucuauguaccuagacaaugcaaaguuaccaucuucuucuuuaagaacagcaauuguuuuuuuuuuaaacaaacugcaucugaaccguuggcuuaauccaa

ggaggguguggccugagaacauagg	2	0	JCK-2
ggaggguguggccugagaacauagg	2	0	JCK-3
ggaggguguggccugagaacauagg	2	0	JCK-1
Aaggguguggccugagaacauagg	1	1	JCK-1
gaggguguggccugagaacauagg	9	0	JCK-3
gaggguguggccugagaacauagg	16	0	JCK-1
gaggguguggccugagaacauagg	11	0	JCK-2
gaggguguggccugagaacauagg	1	0	LT-2
gaggguguggccugagaacauaggA	3	1	JCK-3
gaggguguggccugagaacauaggA	8	1	JCK-1
gaggguguggccugagaacauaggA	3	1	LCK-3
gaggguguggccugagaacauaggA	5	1	JCK-2
Cgguguggccugagaacauagg	2	1	JCK-1
gguguggccugagaacaC	1	1	JCK-3
gguguggccugCgaacau	1	1	JCK-3
gguguggccugagaacau	10	0	JCK-2
gguguggccugagaacau	14	0	JCK-3
gguguggccugagaacau	4	0	LCK-3
ggugugggAcugagaacau	1	1	JCK-2
gguguggccugagaacau	2	0	JCK-1
gguguggccugCgaacau	1	1	JCK-2
gguguggccugagaacau	5	0	LT-2
gguguggccugNgaacau	1	1	JCK-1
gguguggccugagaacau	8	0	LT-3
gguguggccugagaacau	8	0	LT-1
gguguggccugagaacaua	59	0	LCK-3
gguguggccugagaacauC	1	1	JCK-1
gguguggccugagaacauU	2	1	JCK-3
gguguggccugagaacaua	7	0	JT-2
gguguggccugagaacauU	12	1	JT-1
gguguggccugagaacauU	1	1	LT-1
gguguggccugagaacauU	3	1	LT-3
gguguggccugagaCcaua	1	1	LCK-3
gguguggccugagaacaua	25	0	LT-1
gguguggccugagaacauU	7	1	LCK-3
gguguggccugagaacaua	33	0	JCK-3
gguguggccugagaacaua	26	0	LT-3
gguguggccugagaacaua	22	0	LT-2
gguguggcAugagaacaua	1	1	JT-3
gguguggccugagaacaua	8	0	JT-3
gguguggccugagaacauU	3	1	JCK-1
gguguggcAugagaacaua	1	1	JCK-3
gguguggccugagaacaua	56	0	JCK-2
gguguggccugagaacauU	7	1	JCK-2
gguguggccugCgaacaua	1	1	LT-2
gguguggccugagaacaua	46	0	JCK-1
gguguggccugagaacauag	77	0	JCK-1
gguguggccugagaCcauag	1	1	LT-3
gguguggccugagaacauag	17	0	LT-1
gguguggccugagaacauag	82	0	JCK-3
gguguggccugagaacauag	125	0	JCK-2
gguguggccugagaacauag	6	0	LT-2
gguguggccugagaacauaA	1	1	JCK-2
gguguggccugagaacauag	16	0	LCK-3
gguguggccugagaacauag	4	0	JT-2
gguguggccugagaacauag	17	0	LT-3
gguguggccugagaacauag	7	0	JT-3
gguguggccugagaacauaA	1	1	JCK-1
Aguguggccugagaacauagg	1	1	JCK-2
gguguggccugagaacauCgg	1	1	LT-1
gguUggccugagaacauagg	1	1	JCK-2
gguguggccugagaacauagg	20	0	LCK-1
gAuguggccugagaacauagg	1	1	JCK-1
gguguggccugagaacauagg	22	0	JT-2
gguguggccugagaacauagg	916	0	JCK-3
gguguggccugagaacauagA	11	1	JCK-2
gguguggccugagaacauagA	6	1	JCK-3
gguguggccugCgaacauagg	2	1	JCK-3
Uguguggccugagaacauagg	1	1	JCK-3
gAuguggccugagaacauagg	2	1	JCK-3

```
                      Mature                                                  Star
uuaugcagacgguucacccggagguguggccugagaacauagguggguuugcuggcgcgaacucgcucauguaccuuuuguguucucaggucaccccuuugggacacccucuguuuacaugcauauuuuauaugcacaaagcuuucucucucuauguaccuagacaaugcaaaguuaccaucuucuucuuuaagaacagcaauuguuuuuuuuuuaaacaaacugcaucugaaccguuggcuuaauccaa
......................gguguggccugagaacauagg............................................................................................................................................................................................................... 1259 0 JCK-2
......................gguguggccugagaacauagg............................................................................................................................................................................................................... 29 0 JT-3
......................gguguggccugagaacauagU............................................................................................................................................................................................................... 1 1 LT-3
......................gNuguggccugagaacauagg............................................................................................................................................................................................................... 1 1 JCK-1
......................gguguggcAugagaacauagg............................................................................................................................................................................................................... 1 1 JCK-2
......................gguguggccugagaacauagg............................................................................................................................................................................................................... 81 0 LT-2
......................gguguggccugagaacauagA............................................................................................................................................................................................................... 9 1 JCK-1
......................gguguggcUugagaacauagg............................................................................................................................................................................................................... 1 1 JCK-3
......................gguguggccugagaCcauagg............................................................................................................................................................................................................... 1 1 LT-3
......................gguguggccugagaacauagg............................................................................................................................................................................................................... 787 0 JCK-1
......................gguguggccugCgaacauagg............................................................................................................................................................................................................... 1 1 JCK-2
......................gguguggccugagaacauagU............................................................................................................................................................................................................... 33 1 JCK-2
......................gguguggccugagaacauagC............................................................................................................................................................................................................... 5 1 JCK-3
......................gNuguggccugagaacauagg............................................................................................................................................................................................................... 1 1 JCK-2
......................gguguggccugaUaacauagg............................................................................................................................................................................................................... 1 1 JCK-2
......................gguguggccugagaacauagC............................................................................................................................................................................................................... 1 1 JCK-1
......................gguAuggccugagaacauagg............................................................................................................................................................................................................... 1 1 JCK-1
......................gNuguggccugagaacauagg............................................................................................................................................................................................................... 1 1 JT-2
......................gguguggAcugagaacauagg............................................................................................................................................................................................................... 1 1 JCK-2
......................gguguggcAugagaacauagg............................................................................................................................................................................................................... 1 1 LT-3
......................gguguggccugagaacauagg............................................................................................................................................................................................................... 48 0 JT-1
......................gguguAgccugagaacauagg............................................................................................................................................................................................................... 1 1 JCK-1
......................gguguggccugGgaacauagg............................................................................................................................................................................................................... 1 1 LT-3
......................gguguggccugagaGcauagg............................................................................................................................................................................................................... 1 1 JCK-1
......................gguguggccugagaacauagC............................................................................................................................................................................................................... 4 1 JCK-2
......................gAuguggccugagaacauagg............................................................................................................................................................................................................... 2 1 JCK-2
......................gguguggccugagaacauaNg............................................................................................................................................................................................................... 1 1 JCK-1
......................Aguguggccugagaacauagg............................................................................................................................................................................................................... 1 1 LT-2
......................gguguggccugagaaAauagg............................................................................................................................................................................................................... 1 1 JT-3
......................gguguggccugagaacauagg............................................................................................................................................................................................................... 154 0 LT-3
......................gguguggccugagaacauagU............................................................................................................................................................................................................... 2 1 LT-1
......................gguguggccugagaacauagg............................................................................................................................................................................................................... 95 0 LCK-3
......................gguguggccugagaacauagg............................................................................................................................................................................................................... 22 0 LCK-2
......................gguguggccugagaacauagU............................................................................................................................................................................................................... 26 1 JCK-1
......................gguguggccugagaacauagg............................................................................................................................................................................................................... 109 0 LT-1
......................gguguggccugagaacauagN............................................................................................................................................................................................................... 1 1 JCK-1
......................gguguggcUugagaacauagg............................................................................................................................................................................................................... 4 1 JCK-2
......................gguguggccugagaacauagU............................................................................................................................................................................................................... 22 1 JCK-3
......................gguguggccugagaacauaggA.............................................................................................................................................................................................................. 4 1 JCK-2
......................gguguggccugagaacauaggA.............................................................................................................................................................................................................. 1 1 LT-1
......................gguguggccugagaacauaggA.............................................................................................................................................................................................................. 4 1 JCK-3
......................gguguggccugagaacauaggA.............................................................................................................................................................................................................. 1 1 JCK-1
......................gguguggccugagaacauaggug............................................................................................................................................................................................................. 1 0 LCK-3
......................gguguggccugagaacauagguU............................................................................................................................................................................................................. 1 1 LT-1
......................gguguggccugagaacauagguggg........................................................................................................................................................................................................... 2 0 LT-2
.......................gGguggccugagaacauagg............................................................................................................................................................................................................... 1 1 JCK-2
.......................guguggccugagaacauagg............................................................................................................................................................................................................... 1 0 JCK-2
.......................gGguggccugagaacauagg............................................................................................................................................................................................................... 1 1 JCK-3
.......................gGguggccugagaacauagg............................................................................................................................................................................................................... 1 1 JCK-1
........................uguggccugagaacauagg............................................................................................................................................................................................................... 3 0 LT-1
........................uguggccugagaacauagg............................................................................................................................................................................................................... 2 0 JT-3
........................uguggccugagaacauagg............................................................................................................................................................................................................... 7 0 JT-2
........................uguggccugagaacauagg............................................................................................................................................................................................................... 1 0 LT-3
........................uguggccugagaacauagg............................................................................................................................................................................................................... 1 0 LT-2
........................uguggccugagaacauaggu.............................................................................................................................................................................................................. 2 0 LT-1
........................uguggccugagaacauaggug............................................................................................................................................................................................................. 1 0 LT-3
........................uguggccugagaacauaggug............................................................................................................................................................................................................. 5 0 LT-1
........................uguggccugagaacauagguggg........................................................................................................................................................................................................... 2 0 JCK-2
........................uguggccugagaacauagguggg........................................................................................................................................................................................................... 1 0 LT-3
........................uguggccugagaacauagguggg........................................................................................................................................................................................................... 4 0 JCK-3
........................uguggccugagaacauaggugggu.......................................................................................................................................................................................................... 1 0 LT-2
........................uguggccugagaacauaggugggu.......................................................................................................................................................................................................... 1 0 JCK-2
........................uguggccugagaacauaggugggu.......................................................................................................................................................................................................... 1 0 JCK-3
........................uguggccugagaacauaggugggu.......................................................................................................................................................................................................... 1 0 LT-1
........................uguggccugagaacauagguggguu......................................................................................................................................................................................................... 1 0 LT-1
.........................guggccugagaacauagg............................................................................................................................................................................................................... 4 0 LT-2
............................................................................uuuguguucucaggucacccc......................................................................................................................................................... 1 0 LT-1
..............................................................................uguguucucaggucaccccu........................................................................................................................................................ 4 0 JCK-1
..............................................................................uguguucucaggucaccccu........................................................................................................................................................ 2 0 LT-3
..............................................................................uguguucucaggucaccccu........................................................................................................................................................ 2 0 LT-2
```

```
                Mature                                          Star
uuaugcagacgguucacccggagguguggccugagaacauaggugggguuugcuggcgcgaacucgcucauguaccuuuuguguucucaggucaccccuuugggacacccucuguuuacaugcauauuuuauaugcacaaagcuuucucucucuauguaccuagacaaugcaaaguuaccaucuucuucuuuaagaacagcaauuguuuuuuuuuaaacaaacugcaucugaaccguuggcuuaauccaa
...............................................................................uguguucucaggucaccccu.......................................................................................................................................................   4   0   JCK-2
...............................................................................uguguucucaggucaccccu.......................................................................................................................................................   3   0   JCK-3
...............................................................................uguguucucaggucaccccu.......................................................................................................................................................   1   0   LT-1
...............................................................................uguguucucaggucGccccuu......................................................................................................................................................   7   1   JCK-3
...............................................................................uguguucucaggucaccccuu......................................................................................................................................................   44  0   JCK-2
...............................................................................uguguucucaggucGccccuu......................................................................................................................................................   11  1   JCK-2
...............................................................................uguguucucaggucaccccuu......................................................................................................................................................   24  0   JCK-1
...............................................................................uguguucucaggucGccccuu......................................................................................................................................................   1   1   JCK-1
...............................................................................uguguucucaggucaccccAu......................................................................................................................................................   1   1   LT-3
...............................................................................uguguucucaggucaccccuu......................................................................................................................................................   36  0   JCK-3
...................................................................................uucucaggucaccccuuu.....................................................................................................................................................   4   0   LCK-3
...................................................................................uucucaggucaccccuuug....................................................................................................................................................   1   0   JCK-3
...................................................................................uucucaggucCccccuuug....................................................................................................................................................   1   1   JCK-2
...................................................................................uucucaggucaccccuuug....................................................................................................................................................   1   0   JCK-1
```
